# Perinatal Insights Into Parenting, Pathways, and Addiction (PIPPA): Protocol for a Longitudinal, Strengths-Based Study in Flanders, Belgium

**DOI:** 10.2196/88030

**Published:** 2026-05-21

**Authors:** Sarah Vandewalle, Wouter Vanderplasschen, Sara Rowaert, Sarah De Pauw, Gilbert Lemmens

**Affiliations:** 1 Department of Special Needs Education, Faculty of Psychology and Educational Sciences, Ghent University, Henri Dunantlaan 1, Ghent, Flanders, 9000, Belgium, 32 93310310; 2 Department of Head and Skin, Faculty of Medicine and Health Sciences, Ghent University, Ghent, Flanders, Belgium; 3 Department of Psychiatry, Ghent University Hospital, Ghent, Flanders, Belgium

**Keywords:** perinatal substance use, window of opportunity, pregnant and parenting women, substance use disorder, strengths-based study, mixed methods, longitudinal

## Abstract

**Background:**

Perinatal substance use is a growing global health concern with significant risks for maternal health, child development, and parenting. Despite these risks, the perinatal period offers a “window of opportunity” for behavioral change and recovery initiation. However, research exploring how vulnerability and transformation interact in the context of perinatal substance use remains scarce. Existing studies are predominantly cross-sectional, deficit-oriented, and focused on relapse or the medical effects of prenatal exposure, leaving critical gaps in understanding maternal trajectories and psychosocial factors shaping the transition to parenthood.

**Objective:**

The PIPPA (Perinatal Insights into Parenting, Pathways, and Addiction) study identifies the maternal, psychosocial, and contextual factors associated with whether the perinatal period becomes a promising or challenging transition to parenthood for women with substance use problems. Specific outcomes include trajectories of maternal mental health, recovery capital, substance use patterns, mother-child relationship quality, parental stress, and contextual conditions, as well as custody and cohabitation outcomes. An important focus is how these factors shape short- and long-term maternal and child trajectories.

**Methods:**

PIPPA is a longitudinal, prospective, multicenter, mixed methods study in Flanders, Belgium (May 2025-November 2026). Fifty pregnant and parenting women with alcohol and/or other substance use problems are recruited through obstetric departments, general substance use services, specialized substance use services, child protection services, and social media. Participants are assessed at 2-3 points: during pregnancy, 2‐6 weeks postpartum, and 6 months postpartum. Data are collected using validated questionnaires (Alcohol Use Disorder Identification Test-Consumption, Drug Use Disorder Identification Test-Consumption, Edinburgh Postnatal Depression Scale, Satisfaction with Life Scale, Brief Assessment of Recovery Capital-10, Maternal Antenatal Attachment Scale, Maternal Postnatal Attachment Scale, and Parental Stress Scale) and custom instruments. Qualitative data include Three-Minute and Five-Minute Speech Sample tasks to assess maternal representations and expressed emotion, and semistructured interviews with pregnant and parenting women and caregivers. Quantitative analyses will include descriptive, longitudinal, subgroup, and regression models, while qualitative analyses will include longitudinal thematic analysis and structured coding of expressed emotion. All data are managed in REDCap (Research Electronic Data Capture).

**Results:**

Recruitment began in May 2025 and is expected to be complete in November 2026. Data collection began in June 2025. As of April 2026, 13 participants have been recruited. Data analysis will be performed after data collection. The results are expected to be published by the end of 2027.

**Conclusions:**

This study will contribute to a more strengths-based and evidence-informed understanding of the transition to motherhood in the context of perinatal substance use. The PIPPA study will capture diverse experiences and the complex interplay among substance use, recovery, and early parenting. These insights will inform and strengthen integrated, responsive early interventions to support pregnant and parenting women with substance use problems and promote child well-being.

## Introduction

### Background

Substance use among pregnant and parenting women is increasingly recognized as a growing global public health concern [1,2]. Prevalence rates vary considerably across studies, shaped by sociodemographic characteristics, national legislation, and the reliability of self-reporting and disclosure practices [3,4]. These variations, compounded by methodological challenges and underreporting, make it difficult to establish accurate estimates [5,6]. Nonetheless, recent data suggest that substance use during pregnancy affects 4%-15% of all women [3]. In the United States, approximately 6% of women use illicit substances during pregnancy [7], and about 10% consume alcohol while pregnant [8]. Additionally, nearly 1 in 10 children younger than 6 years lives with an adult who has a substance use disorder (SUD) [9,10]. In Europe, substance use during pregnancy is reported in 6.5%‐11% of cases [11], while the prevalence of parental SUD ranges from 6.2% to 35.2% [12]. In Belgium, a recent study found that 14.6% of women consume alcohol, 4% use tobacco, and 0.4% use illicit substances during pregnancy, with substantial variation across trimesters [13]. Accurate data on parental substance use remain unavailable due to major data gaps and fragmented registration systems. However, recent figures indicate that approximately 22% of individuals entering treatment live with minors, although it is unclear whether these minors are their biological children, their partner’s children, or younger siblings [14]. Furthermore, a 2015 study suggested that 12% of children live with parents who misuse alcohol, while fewer than 1% live with parents who use illicit drugs [15].

The risks associated with substance use during the perinatal period are multifaceted and far-reaching. Physiologically, teratogenic risks are well documented. Most substances cross the placental barrier, affecting the uterus, the placenta, and fetal development [16]. Psychosocially, substance use problems are often associated with parenting challenges. Mothers with substance use problems often exhibit lower sensitivity and more passive, harsh, or disengaged behaviors during interactions with their infants, and they often lack essential parenting knowledge and have inappropriate development expectations [17,18]. Difficulties in forming a healthy parent-child relationship are common and may be partly explained through the neurobiological overlap between SUD and attachment systems [18]. Moreover, other factors often contribute to poor parenting behaviors, including maternal childhood trauma, co-occurring mental health disorders, exposure to intimate partner violence, increased vulnerability to parenting stress, homelessness, and a lower socioeconomic status [17-20].

Beyond the developmental and relational consequences for the child, substance use during the perinatal period also poses substantial risks to maternal health and well-being. Pregnancy increases vulnerability to interpersonal violence due to heightened physical, emotional, and financial demands [21]. Overdose has become one of the leading causes of preventable death among postpartum women with substance use problems, contributing to a tripling of drug-related pregnancy-associated mortality in recent years [22,23]. Moreover, since the perinatal period may also be a source of increased stress [24]—physical, psychological, and societal—many women continue to use substances during pregnancy [25], and relapse among those who initially reduce or cease use is most common in the third trimester [26] or early postpartum period [25,27-30]. Notably, research has suggested that up to 80% of women who are abstinent in the last month of pregnancy relapse within the first 2 years postpartum [28].

These risks often result in child welfare involvement. Over the past 2 decades, parental SUD has become a major factor in out-of-home placements [31,32]. It is estimated that between 50% and 80% of children in foster care have at least one parent with SUD [33]. However, maternal and infant trajectories vary widely. Some mothers retain custody, others experience separation, and some are later reunited. The relationship between maternal SUD and child removal is not straightforward [34].

Still, the perinatal period is characterized by high motivation to initiate behavioral change, seek treatment, and engage in recovery [11,17,35]. After all, many women cease or reduce their substance use during pregnancy [28,30,36]. Their motivations are rooted in the desire to ensure a healthy pregnancy and birth outcome, improve personal well-being, and avoid child welfare involvement [9,30,31,35]. The perinatal period thus represents a critical phase of growth and transformation [30] and is often described as a “window of opportunity” [21,27,37,38]. For caregivers, the increased frequency and intensity of contact with health care and support services during this time offer a unique opportunity to build trust and establish long-term care trajectories [9,39]. However, despite this potential for positive change, navigating the perinatal period is particularly demanding for these women. In addition to the physical, emotional, and psychological changes experienced by all pregnant and postpartum women, they must manage their own addiction and/or recovery while dealing with the challenges of pregnancy or parenting, which may be further complicated by the effects of prenatal substance exposure and the scrutiny of legal, social, and moral systems [2,40].

### Aims of the Study

This paradox of the perinatal period encompassing both vulnerability and potential transformation underscores the need for a deeper understanding. Yet, research examining how these dynamics interact in the context of perinatal substance use remains scarce. The phenomenon is often concealed due to stigma and underreporting [41], and most information is derived from cross-sectional surveys and retrospective reports [28]. The limited prospective studies available primarily focus on the relapse process in this period or the medical effects of prenatal substance exposure on the child [28,33,42], and have generally adopted a deficit-based approach. Few studies have systematically examined the trajectories of women navigating both motherhood and substance use, specifically exploring how they make meaning of experiences and the factors that contribute to a successful transition to parenthood among women with substance use problems.

The PIPPA (Perinatal Insights into Parenting, Pathways, and Addiction) study (May 2025 to November 2026) aims to address this gap through a longitudinal observational design that adopts a strengths-based approach, which emphasizes that individuals possess capacities, resources, and potential for change, even in the context of significant adversity [43]. This approach aligns with the recovery paradigm in addiction, which highlights connectedness, hope, personal agency, meaning, and empowerment as key elements supporting the recovery process [44,45].

Conducted in Flanders, the Dutch-speaking region of Belgium, the study follows pregnant and parenting women with substance use problems throughout the perinatal period. It examines perinatal psychosocial factors, contextual influences, and recovery capital to understand how these elements relate to parental development and the evolving trajectories of both mother and child.

The central research question is as follows: Which maternal, psychosocial, and contextual factors influence whether the perinatal period represents a promising or challenging transition to parenthood for pregnant and parenting women with substance use problems, and how do the trajectories of mother and child unfold in the short and long term? Additional subquestions include the following: (1) What are the sociodemographic, substance use, pregnancy, parenthood, and psychosocial characteristics of women with substance use problems, and how do they describe their experiences during the perinatal period? (2) How do maternal mental health, recovery capital, substance use, and mother-child relationship quality evolve across the perinatal period, and how are these trajectories related to substance use severity? (3) Which maternal, psychosocial, and behavioral factors predict custody retention, cohabitation, and recovery opportunities during the perinatal period?

## Methods

### Study Design

The PIPPA study is a longitudinal, prospective, multicenter, mixed methods study following pregnant and parenting women with substance use problems throughout the perinatal period. For the purpose of this protocol, the topic is limited to pregnant and parenting women; this terminology is not intended to exclude or overlook the experiences of people of other genders who may also experience pregnancy and parenthood.

**Figure 1.**
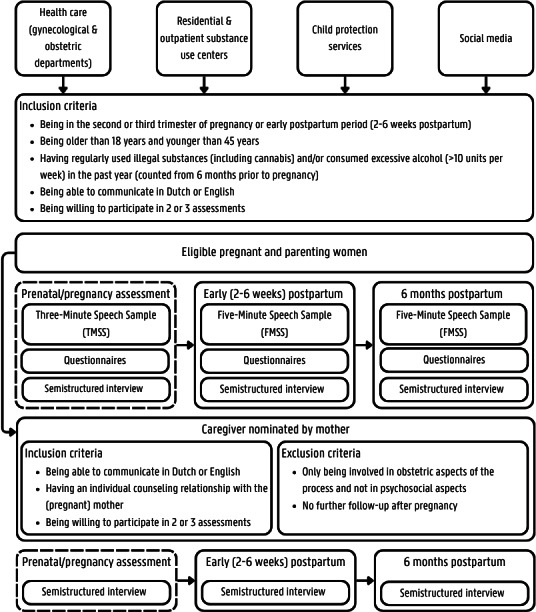
Study design.

Participants are assessed at 2-3 points: during pregnancy, between 2 and 6 weeks postpartum, and at 6 months postpartum (Figure 1). In cases where mothers are enrolled after giving birth, the prenatal assessment is logically omitted.

### Participants and Settings

The PIPPA study aims to gain more insight into the transition to motherhood for pregnant and parenting women with alcohol and/or other substance use problems by exploring the maternal, psychosocial, and contextual factors that shape their experiences during pregnancy and the first 6 months of motherhood. Given the sensitivity of the topic and the fragile circumstances of many of these women, we aim to recruit 50 pregnant and parenting women within 1.5 years, with a follow-up period of 6 months. This sample size was determined in consultation with the various centers that provide services to this hard-to-reach population and is expected to provide adequate power for descriptive, longitudinal, subgroup, and regression analyses, depending on the specific research question and statistical method (see the Data Analysis Plan section).

Participants are recruited across Flanders through multiple service sectors (Table 1). Recruitment takes place in clinical settings, specifically the gynecological and obstetric departments of 7 general and university hospitals. In addition, both residential and outpatient substance use treatment centers are involved. These include general substance use services, such as 3 low-threshold multidisciplinary *Medisch-Sociaal OpvangCentra* (low-threshold harm-reduction centers), which focus on harm reduction and provide medical care, psychosocial support, and social reintegration, as well as *Verslavingskoepel vzw*, where experts by experience voluntarily provide support and guidance together with professional care providers. Moreover, several specialized initiatives that specifically target pregnant and parenting women with substance use problems are involved. Two of these facilities, *Antwerps Drug Interventie Centrum – Ontwenningsprogramma ouders en kind* (Antwerp Drug Intervention Center *–* Detoxification program for parents and children) and *De Kiem* (Therapeutic community, Tipi), provide residential treatment, while others, such as *vzw Kompas* (*Drugspecifieke thuisbegeleiding voor gebruikende ouders met jonge kinderen* [Substance-specific home-based support for substance-using parents with young children]) and *Free Clinic* (*Gezinnen onder invloed Antwerpen* [Families under the influence Antwerp]), offer outreach and ambulatory support. Furthermore, 2 perinatal support networks (*Perinataal Ondersteuningsnetwerk Druggebruikende Ouders* [Perinatal support networks for drug-using parents]) participate in the recruitment. These initiatives bring together health care, substance use, and child protection services to support pregnant and parenting women who use substances, with the primary aim of safeguarding the child’s well-being through early, coordinated, and family-centered care. Additional recruitment occurs through child protection services in the Flemish provinces of East and West Flanders, which are mandated to safeguard children’s well-being and may intervene when parental substance use poses risks to children’s safety. Finally, participants are also recruited through social media campaigns on Facebook, LinkedIn, and Instagram, which inform both caregivers and pregnant and parenting women about the study’s aims, inclusion criteria, and contact options (email, phone, QR code, or direct message).

**Table 1.** Overview of recruitment sources.

Recruitment source and type of service		Organization/initiative
Health care		
Gynecological and obstetric departments		7 general and university hospitals
Substance use services		
General services		*Verslavingskoepel vzw,* 3 MSOC^a^ centers, and *De Kiem* (outreach centers)
Specialized services for pregnant and parenting women		Residential: ADIC (OP+)^b^ and *De Kiem* (therapeutic community, Tipi); Outreach/ambulatory: *vzw Kompas* (DODO^c^) and *Free Clinic* (GoiA^d^); Two perinatal support networks (PONDO^e^)
Child protection		
Provincial services		East & West Flanders Child Protection Services
Digital outreach		
Social media		Facebook, Instagram, and LinkedIn

a MSOC: *Medisch-Sociaal OpvangCentra* (low-threshold harm-reduction centers).

b ADIC (OP+):* Antwerps Drug Interventie Centrum – Ontwenningsprogramma ouders en kind *(Antwerp Drug Intervention Center *–* Detoxification program for parents and children).

c DODO: *Drugspecifieke thuisbegeleiding voor gebruikende ouders met jonge kinderen* (Substance-specific home-based support for substance-using parents with young children).

d GoiA: *Gezinnen onder invloed Antwerpen* (Families under the influence Antwerp).

e PONDO: *Perinataal Ondersteuningsnetwerk Druggebruikende Ouders* (Perinatal support networks for drug-using parents).

The eligibility criteria for pregnant and parenting women include (1) being in the second or third trimester of pregnancy or in the early postpartum period, (2) being older than 18 years and younger than 45 years, (3) having regularly used illegal substances (including cannabis) and/or consumed excessive alcohol (>10 units per week) [46,47] in the past year (counted from 6 months prior to pregnancy), (4) being able to communicate in Dutch or English, and (5) being willing to participate in 2-3 assessments. During pregnancy or shortly after birth, women are informed about the aims and design of the PIPPA study through flyers and by staff members of the collaborating facilities. At the initial meeting with the researcher, they receive detailed information about the study and the implications of study participation and are asked to provide written informed consent.

Participants are also asked if an involved professional caregiver who plays an important role in their trajectory may be contacted. With the participant’s consent, this caregiver is approached for a semistructured interview at each assessment point. The eligibility criteria for these caregivers include (1) being able to communicate in Dutch or English, (2) having an individual counseling relationship with the pregnant and parenting women, and (3) being willing to participate in 2-3 assessments. The exclusion criteria are (1) being involved only in the obstetric aspects of the process and not in the psychosocial aspects, and (2) having no further follow-up after pregnancy.

### Participant Engagement and Retention: A Socioecological Approach

Working with a hard-to-reach and often vulnerable population presents several methodological and practical challenges. The process of recruiting and retaining participants is particularly complex due to challenging life circumstances; existing stressors of pregnancy; multiple appointments with care providers; and barriers such as limited mobility, fear of stigma, and custody concerns [48,49]. To address these difficulties, this study incorporates recommendations for recruiting and retaining pregnant women in longitudinal research through the following five levels of socioecological influence [48,49]:

1. Individual level: Respectful, nonjudgmental, and compassionate engagement; flexible scheduling and rescheduling; autonomy in choosing the study location; option of home visits; comprehensible and comprehensive information; efforts to reduce transport and childcare barriers [50]; and financial compensation in the form of a gift card for time and effort.
2. Interpersonal level: All study visits conducted by the same researcher to ensure continuity, consistency, and familiarity for pregnant and parenting women; and introductory visits to centers, clear and concise communication, and monthly reminders to sustain engagement while minimizing burden for care providers.
3. Organizational level: Data collection conducted in conjunction with regular appointments and embedded within perinatal care and addiction services to enhance inclusion and follow-up opportunities, foster trust, and minimize participant burden [49,51]; data collection duration (75‐90 min) within the recommended timeframe; inclusion of caregiver interviews to ensure continuity when maternal dropout occurs; and provision of referral options and the contact information of a perinatal psychiatrist when difficult emotions are experienced following participation [49].
4. Community level: Sustained collaboration with involved organizations through frequent mailings, in-person discussions, and introductory visits; use of connections established with several centers through previous recruitment efforts for prior studies, fostering trust and facilitating collaboration; and outreach through social media platforms targeting caregivers and through groups specifically designed for individuals in recovery and pregnant and parenting women [48].
5. Policy level: Transparent discussion of reporting procedures in a context without compulsory prenatal child-protection measures, thereby balancing participant autonomy with safety considerations: “If any concerns regarding your wellbeing/health, or the wellbeing/health of your child, are noticed during the follow-up, they will first be discussed with you, and it will be suggested to further address these concerns with the hospital’s social services, your general practitioner, or another involved party. If serious concerns arise regarding a genuine, serious, and immediate danger to you or your child, the researcher may, after consulting her supervisors (eg, [name] perinatal psychiatrist), and if deemed necessary, also contact the helpline 1712 to further discuss the situation.”

This multilevel strategy is designed to enhance recruitment, reduce drop-out, and maximize feasibility in a complex and vulnerable population.

### Study Procedure

#### Prenatal Assessments

Potential participants are first screened according to the inclusion criteria. Eligible women then complete the Three-Minute Speech Sample task (TMSS) (see the Instruments section), in which they are invited to talk freely about their unborn child and their relationship with the child. This task provides insights into maternal representations, feelings, and prenatal attachment. A short semistructured interview further explores the mother’s general well-being, the development and health of the unborn child, the expectations of future parenthood, substance use (including cannabis and alcohol) in the context of pregnancy, and the role of interpersonal relationships and the social environment in influencing substance use (Multimedia Appendix 1).

Furthermore, participants complete questionnaires on sociodemographic characteristics, substance use and treatment history, pregnancy and parenthood, contextual factors (formal and informal support and partner relationship), recovery capital, perinatal mental health, life satisfaction, and the prenatal mother-child relationship.

A researcher (SV) conducts the interviews and completes the questionnaires by using REDCap (Research Electronic Data Capture), a secure, web-based platform widely used for academic and clinical research data management. Each session takes approximately 75‐90 minutes to complete, after which participants receive a €15 (US $17.47) voucher. Following the prenatal assessment, 2 postnatal appointments are scheduled based on the estimated date of delivery.

#### Postnatal Assessments

For mothers enrolled in the study after childbirth, screening for the inclusion criteria and collection of sociodemographic information are performed at the first postnatal assessment (2‐6 weeks postpartum). The second postnatal assessment takes place 6 months postpartum. To support retention, participants are contacted approximately 2 weeks before each appointment by phone or SMS text message reminder.

At both postnatal assessments, the Five-Minute Speech Sample task (FMSS) is administered (see the Instruments section), in which mothers are invited to talk freely about their child and their relationship with the child, providing insights into maternal representations and the quality of the early mother-child bond. Furthermore, a brief semistructured interview explores the mother’s general well-being, the child’s health and development, experiences of parenthood, substance use (including cannabis and/or alcohol) in the context of parenting, and the influence of interpersonal and social environments on substance use (Multimedia Appendix 1). Participants also complete questionnaires on substance use (type of substance and frequency in the past 30 days) and treatment, contextual factors (formal and informal support networks and partner relationship), recovery capital, perinatal mental health, life satisfaction, the postnatal mother-child relationship, and parental stress. Each postnatal session takes approximately 75‐90 minutes, and participants again receive a €15 (US $17.47) voucher for each completed assessment.

#### Interview With the Involved Professional Caregiver

At each assessment point, a caregiver chosen by the mother is invited to participate in a semistructured interview. This interview explores the caregiver’s perspective on the mother’s well-being, the development and health of the child, the mother’s experiences of pregnancy and parenthood, substance use in the context of parenting, and the influence of interpersonal and social environments on substance use (Multimedia Appendix 2). The interviews last approximately 30‐45 minutes and complement the maternal assessments.

### Instruments

#### Overview

To comprehensively assess maternal, substance use, psychosocial, and contextual factors across the perinatal period, a combination of custom and validated instruments is used. These tools capture sociodemographic characteristics, substance use patterns, recovery capital, mental health, life satisfaction, and parent-child relationship quality. An overview of all instruments and assessments is provided in Table 2, followed by a detailed description of each measure, including custom questionnaires, validated scales (eg, Drug Use Disorder Identification Test-Consumption [DUDIT-C], Alcohol Use Disorder Identification Test-Consumption [AUDIT-C], Brief Assessment of Recovery Capital-10 [BARC-10], Edinburgh Postnatal Depression Scale [EPDS], Satisfaction with Life Scale [SWLS], Maternal Antenatal Attachment Scale [MAAS], Maternal Postnatal Attachment Scale [MPAS], and Parental Stress Scale [PSS]), and speech samples (TMSS and FMSS).

**Table 2.** Overview of instruments and assessments.

Category and instrument			Topic	Prenatal assessment	Postnatal assessment at 2‐6 weeks	Postnatal assessment at 6 months
Mother						
Baseline substance use						
AUDIT-C^a^			Alcohol use	✓	✓^b^	—^c^
DUDIT-C^d^			Substance use	✓	✓^b^	—
Contextual and background measures						
Sociodemographic			Age^b^, educational level^b^, marital status^b^, employment status, and living situation	✓	Living situation	Employment status and living situation
Substance use			Past year and prepregnancy use^b^, past 30 days use, changes in postpregnancy use^b^, treatment history^b^, and current treatment	✓	Past 30 days substance use and current treatment	Past 30 days substance use and current treatment
Pregnancy and parenthood			Number of children plus custody status, number of pregnancies, number of miscarriages, number of abortions, planned/unplanned pregnancy, discovery of pregnancy, and consideration of abortion	✓	✓^b^	—
Contextual factors			Informal and formal support, and substance use partner	✓	✓	✓
Assessment instruments						
BARC-10^e^			Recovery capital	✓	✓	✓
EPDS^f^			Perinatal depression	✓	✓	✓
SWLS^g^			Life satisfaction	✓	✓	✓
MAAS^h^			Prenatal mother-child relationship	✓		
MPAS^i^			Postnatal mother-child relationship	—	✓	✓
PSS^j^			Parental stress	—	✓	✓
TMSS^k^			Emotional climate of the parent-child relationship	✓	—	—
FMSS^l^			Emotional climate of the parent-child relationship	—	✓	✓
Interview			Well-being of the mother, development and health of the child, parenthood or future parenthood, substance use in combination with pregnancy/parenthood, and influence of relationships/social environments	✓	✓	✓
Involved professional caregiver						
Interview			Well-being of the mother, development and health of the child, parenthood or future parenthood, substance use in combination with pregnancy/parenthood, and influence of relationships/social environments	✓	✓	✓

a AUDIT-C: Alcohol Use Disorder Identification Test-Consumption.

b Asked at postnatal assessment (2-6 weeks) if participants are enrolled after giving birth.

c Not applicable.

d DUDIT-C: Drug Use Disorder Identification Test-Consumption.

e BARC-10: Brief Assessment of Recovery Capital-10.

f EPDS: Edinburgh Postnatal Depression Scale.

g SWLS: Satisfaction with Life Scale.

h MAAS: Maternal Antenatal Attachment Scale.

i MPAS: Maternal Postnatal Attachment Scale.

j PSS: Parental Stress Scale.

k TMSS: Three-Minute Speech Sample task.

l FMSS: Five-Minute Speech Sample task.

#### Contextual and Background Questionnaires

Baseline sociodemographic characteristics (eg, age, education level, marital status, work situation, and current living situation), substance use patterns (type and frequency over the past year and past 30 days, and treatment history), and pregnancy and parenthood characteristics (number of children and pregnancies, previous abortions and/or miscarriages, and planned or unplanned pregnancy), as well as contextual factors, such as informal family/social support, formal support, and partner substance use (past year and past 30 days), are assessed using custom questionnaires developed for this study. These measures have been designed to capture relevant information for this population and include both closed-ended questions (eg, multiple choice, Likert-type scales) and open-ended questions to obtain detailed, context-specific data. While not standardized, these instruments provide essential descriptive information to characterize the study sample and its contextual influences.

#### Assessment Instruments

##### DUDIT-C Scale

The DUDIT-C is a shortened version of the DUDIT, and it consists of 4 items capturing the frequency of drug use, the frequency of using more than one substance at the same time, the frequency of substance use on a typical day of substance use, and the frequency of being heavily influenced by substances. The scale is used to screen for problematic substance use [52]. The DUDIT-C is scored on a scale of 0‐16, with a score of 0 reflecting no substance use. For women, a score of ≥2 is considered positive for potential substance use problems.

##### AUDIT-C Scale

The AUDIT-C is a shortened version of the AUDIT screening list, and it consists of 3 items aimed at identifying alcohol misuse. Questions capture the frequency of alcohol use, the quantity of alcohol use on a typical day of alcohol use, and the frequency of 6 or more consumptions of alcohol in the past year [53]. The AUDIT-C is scored on a scale of 0‐12, with a score of 0 reflecting no alcohol use. For women, a score of ≥3 is considered positive for potential alcohol misuse. Generally, a higher score is associated with a greater probability of alcohol consumption impacting the participant’s health and safety [54].

##### BARC-10 Questionnaire

The BARC-10 is a strengths-based, validated questionnaire derived from the 50-item measure of recovery capital (Assessment of Recovery Capital-10). The BARC-10 assesses the level of broader personal, social, physical, and professional resources in an individual’s environment that are used to initiate and sustain recovery, including structural supports, such as recovery-supportive living spaces and community relationships, through 10 questions that measure 10 domains of recovery capital. Answers are rated on a 6-point scale ranging from 1 (strongly disagree) to 6 (strongly agree). The total score can range from a minimum of 10 to a maximum of 60. The internal consistency of the BARC-10 has been found to be good in a clinical sample of service users receiving treatment for SUDs (Cronbach α=0.90) [55].

##### EPDS Assessment

The EPDS is a 10-item self-report scale designed to assess depressive symptoms in the past 7 days, with 1 item assessing suicidal ideation [56]. The EPDS is the most used screening instrument for perinatal depression and is validated for both antenatal and postnatal use. Each item is scored from 0 to 3, and the total score ranges from 0 to 30, with higher scores indicating greater depressive symptom severity. The EPDS can be completed in about 5 minutes. The internal consistency of the Dutch version has been shown to be good in a population of postpartum women (Cronbach α=0.82) [57]. The internal consistency values (Cronbach α) antenatally for the first, second, and third trimesters have been reported to be 0.82, 0.83, and 0.84, respectively [58].

##### SWLS Assessment

The SWLS is a widely used 5-item self-report tool measuring global life satisfaction, a cognitive component of subjective well-being [59]. The SWLS scores are associated with a wide range of personal factors. For instance, scores have been reported to be positively correlated with measures of happiness and quality of life, and negatively correlated with aggression, stress, and feelings of hopelessness [60]. Respondents rate their agreement with each item on a 7-point Likert scale (1 [strongly disagree] to 7 [strongly agree]). The total score is obtained by summing the responses of all 5 items, with higher scores indicating greater life satisfaction. Scores can be interpreted in terms of *relative* life satisfaction by comparing individual scores with scores from normative samples, as well as in *absolute* terms (ie, 5‐9, extremely dissatisfied; 10‐14, dissatisfied; 15‐19, slightly dissatisfied; 20, neutral; 21‐25, slightly satisfied; 26‐30, satisfied; 31‐35, extremely satisfied). The SWLS is reported to have good internal consistency, with a Cronbach α value of 0.87, and has been previously used in research on people with SUDs [61].

##### MAAS Assessment

The MAAS [62] consists of 19 items divided over the following 2 subscales: “quality of attachment” (quality) and “time spent in attachment mode” (preoccupation). All items are scored on a 5-point scale. The minimum score for the total MAAS is 19, and the maximum score is 95. The scores for the quality and preoccupation subscales theoretically range from 11 to 50 and 8 to 40, respectively. High quality and preoccupation scores reflect a positive quality of attachment and a high intensity of preoccupation with the unborn child, respectively. The internal consistency values (Cronbach α) of the Dutch version of the total MAAS for the first, second, and third trimesters have been reported to be 0.79, 0.80, and 0.78, respectively [63].

##### MPAS Assessment

The MPAS [64] consists of 19 items divided over the following 3 subscales: “quality of attachment” (quality), “absence of hostility” (hostility), and “pleasure in interaction” (pleasure). Each item has a 2-, 4-, or 5-point scale response option. To ensure equal weighting of all questions, all response options are recoded to represent a score of 1 (low attachment) to 5 (high attachment). The minimum score for the total MPAS is 19, indicating a problematic mother-to-infant bond, and the maximum score is 95. The scores for the quality and hostility/pleasure subscales theoretically range from 9 to 45 and 5 to 25, respectively, and higher scores reflect a positive quality of attachment and a high intensity of preoccupation with the unborn child, respectively. The internal consistency (Cronbach α) of the Dutch version of the total MPAS has been reported to range from 0.68 to 0.79 [63].

##### PSS Assessment

The PSS [65] is an 18-item self-report scale that assesses perceived parental stress through positive and negative aspects of parenthood. Responses are rated on a 5-point scale ranging from 1 (strongly disagree) to 5 (strongly agree). The scale can be completed in less than 10 minutes. Overall scores range from 18 to 90, with higher scores indicating higher levels of parental stress. The Dutch translation of the PSS is a 15-item questionnaire, with an overall score range from 15 to 75, and the scale has been shown to have good internal consistency (Cronbach α=0.87) [66].

##### FMSS and TMSS

The FMSS is a valid, efficient, and cost-effective method for assessing the emotional climate of the parent-child relationship (expressed emotion) [67,68]. In the FMSS, caregivers are asked to speak for 5 uninterrupted minutes about the child and their relationships. In the TMSS, pregnant women are asked to speak for 3 uninterrupted minutes about their expectations and hopes for their child and their relationships [69]. The TMSS and FMSS are audio-recorded and coded. The TMSS is coded using the FMSS coding system. Both interviews measure 2 distinct components of expressed emotion, namely criticism and emotional overinvolvement (EOI), that are subsumed under the more general labels of high expressed emotion and low expressed emotion. Several subprofiles of expressed emotion exist, namely high criticism, high EOI, high criticism/EOI, and low EOI [70]. Criticism refers to dislike or disapproval of the child’s behavior and is measured by analyzing the initial statement a parent makes about the child and comments that contain disapproval, dislike, or annoyance expressed in content or tone. EOI consists of emotional display, statements of attitude, self-sacrificing or overprotective remarks, excessive detail, and excessive praise.

### Data Analysis Plan

#### Overview

The study uses a convergent parallel mixed-methods design. Quantitative and qualitative data are collected simultaneously at each assessment point, pseudonymized by assigning each participant a unique code, analyzed separately, and subsequently integrated to generate a comprehensive interpretation of maternal and child trajectories. This design, framed within a case-study approach, allows for the examination of measurable patterns while capturing lived experience, nuance, and meaning [71].

Given the vulnerability of the study population, attrition across follow-up assessments is anticipated. Missing quantitative data will be handled using an available-case approach, whereby analyses are conducted on observed data without imputation. This approach is consistent with the mixed-methods case-study design, in which quantitative trajectories are complemented by qualitative data to contextualize individual patterns over time.

The overarching objective is to examine which maternal, psychosocial, and contextual factors influence whether the perinatal period represents a promising or challenging transition to parenthood for women with substance use problems, and how the maternal and child trajectories unfold over the short and long term. Specific outcomes of interest include trajectories of maternal mental health, recovery capital, substance use patterns, mother-child relationship quality, parental stress, contextual factors, and custody/cohabitation outcomes.

#### Research Questions and Analytical Approaches

Research question A is as follows: What are the sociodemographic, substance use, pregnancy, parenthood, and psychosocial characteristics of women with substance use problems, and how do they describe their experiences during the perinatal period? The analyses are as follows:

- Quantitative analyses: descriptive statistics, rank-order stability (Pearson correlations), and mean-level change (repeated-measures ANOVA)
- Qualitative analyses: reflexive thematic analysis (TA) of maternal and caregiver interviews to examine how women experience these characteristics, and how meaning-making and contextual factors shape the perinatal experience

Research question B is as follows: How do maternal mental health, recovery capital, substance use, and mother-child relationship quality evolve across the perinatal period, and how do these trajectories relate to substance use severity? The analyses are as follows:

- Quantitative analyses: subgroup analyses (1-way ANOVA) based on AUDIT-C and DUDIT-C scores
- Qualitative analyses: longitudinal reflexive TA with a trajectory approach, using within-case and cross-case matrices to identify temporal shifts in meanings, concerns, relational experiences, and recovery-related processes

Research question C is as follows: Which maternal, psychosocial, and behavioral factors predict custody retention, cohabitation, and recovery opportunities during the perinatal period? The analyses are as follows:

- Quantitative analyses: regression models exploring the predictors of custody/cohabitation outcomes and recovery opportunities, based on psychosocial, behavioral, and contextual variables across time points
- Qualitative analyses: longitudinal reflexive TA with a trajectory approach, using within-case and cross-case matrices to identify temporal shifts in meanings, concerns, relational experiences, and recovery-related processes that help explain how and why custody outcomes diverge

A summary of quantitative analyses, expected effect sizes, and sample requirements is provided in Table 3.

**Table 3.** Planned statistical analyses with corresponding outcomes, effect sizes, sample requirements, and research questions.

Analysis type	Outcome	Effect size detectable	Number	Alpha value	Power	Research question
Pearson correlation	Rank-order stability	*r*=0.40	46	.05	0.80	A^a^
Repeated measures ANOVA	Mean-level stability	*f*=0.20	42 (3 assessments) 52 (2 assessments)	.05	0.80	A
Subgroup ANOVA (4 groups)	Psychosocial outcomes	*f*=0.50	48	.05	0.80	B^b^
Subgroup ANOVA (2 groups)	Psychosocial outcomes	*f*=0.40	52	.05	0.80	B
Regression (3 predictors)	Custody outcomes	*f*^2^=0.25	48	.05	0.80	C^c^
Regression (2 predictors)	Custody outcomes	*f*^2^=0.20	52	.05	0.80	C

a Research question A: What are the sociodemographic, substance use, pregnancy, parenthood, and psychosocial characteristics of women with substance use problems, and how do they describe their experiences during the perinatal period?

b Research question B: How do maternal mental health, recovery capital, substance use, and mother-child relationship quality evolve across the perinatal period, and how do these trajectories relate to substance use severity?

c Research question C: Which maternal, psychosocial, and behavioral factors predict custody retention, cohabitation, and recovery opportunities during the perinatal period?

#### Descriptive Analyses

This study will be the first longitudinal and in-depth study to follow up on this specific population of women. Many analyses will therefore be mainly descriptive in nature (eg, means and percentages). We emphasize that the study focuses on an underrepresented population that is often marginalized and receives little attention in scientific research, making these descriptive analyses crucial for gaining insights into the complexity of their situation.

#### Longitudinal Analyses

Longitudinal analyses will be restricted to participants with data available for at least two time points and will assess trajectories of maternal mental health, recovery capital, substance use, and mother-child relationship quality across prenatal and postnatal assessments using Pearson correlations (rank-order stability) and repeated-measures ANOVA (mean-level change).

Rank-order stability will be assessed using the Pearson correlation coefficient to determine the consistency of individual differences across time points. Power calculations indicate that a large correlation (*r*=0.40) can be detected with 46 participants (*α*=.05; power=0.80).

Mean-level stability will be evaluated using repeated-measures ANOVA to test for significant changes in group means over time. Power calculations indicate that a small to medium effect size (*f*=0.20) can be detected with a sample of 42 participants (*α*=.05; power=0.80) for 3 assessments and with 52 participants for 2 assessments.

In cases of low correlation, statistical significance may be difficult to determine due to sample size limitations. However, prior research [72] suggests medium-to-large effect sizes in the following similar perinatal contexts:

- Attachment correlations between the second and third trimesters and postnatal period (*r*=0.29 and *r*=0.40, respectively; *P*<.01)
- Mental health correlations across pregnancy trimesters and postnatally (*r*=0.42, *r*=0.52, and *r*=0.60; all *P*<.01)
- Alcohol use correlations across pregnancy trimesters and postnatally (*r*=0.41, *r*=0.30, and *r*=0.48; all *P*<.01)

#### Subgroup Analyses Based on Substance Use Severity

We will examine the extent to which the severity of substance use is associated with psychosocial changes and opportunities during the transition to motherhood. We will incorporate substance use severity as a key independent variable alongside temporal effects. Specifically, we aim to assess whether baseline substance use patterns are predictive of longitudinal changes in recovery capital, mental health, pre- and postnatal attachment, parental stress, and continued substance use. Alcohol use severity will be assessed using the AUDIT-C, and drug use severity will be assessed using the DUDIT-C, with both administered during the first assessment.

In accordance with guidelines from the Belgian Superior Health Council, a threshold of ≥10 alcoholic drinks per week is considered indicative of elevated health risk, with additional recommendations to abstain from alcohol preconceptionally [46,47]. Recognizing the heterogeneity within this population—ranging from episodic heavy use to chronic dependence—these instruments will facilitate subgroup classification based on use patterns.

Participants will be categorized into the following 4 groups based on AUDIT-C and DUDIT-C scores: group A, score of ≥2 on the DUDIT-C; group B, score of ≥3 on the AUDIT-C; group C, score of ≥2 on the DUDIT-C and ≥3 on the AUDIT-C; and group D, score of <2 on the DUDIT-C and <3 on the AUDIT-C. If subgroup sample sizes are insufficient for reliable analysis, groups A, B, and C will be collapsed into a single elevated-use group (AUDIT-C ≥3 and/or DUDIT-C ≥2), and this group will be compared against group D (no elevated score). Group comparisons will be conducted using 1-way ANOVA to test for differences in mean scores across the identified subgroups. In cases where assumptions of normality or homogeneity of variance are violated, the nonparametric Kruskal-Wallis test will be used.

Power calculations indicate that a large effect size (*f*=0.50) can be detected across 4 groups with a sample of 48 participants (*α*=.05; power=0.80), and a medium-to-large effect size (*f*=0.40) can be detected between 2 groups with 52 participants (*α*=.05; power=0.80). Previous research supports the presence of substantial associations between substance use severity and psychosocial outcomes (stress and alcohol use: B=0.47, 95% CI 0.22‐0.73; mental health and alcohol use: B=0.74, 95% CI 0.45‐1.03 for anxiety; B=0.54, 95% CI 0.27‐0.82 for depression) [73].

#### Regression Analyses: Custody and Parenting Outcomes

Regression models will be used to investigate which psychosocial and behavioral factors—such as recovery capital, mental health, mother-child relationship quality, substance use, and parental stress—are associated with custody retention and recovery opportunities during the perinatal phase. This analysis aims to identify the predictors of maternal-child cohabitation outcomes and to explore the broader relational and psychological context in which these outcomes occur. Participants will be categorized into the following 3 groups based on the mother-child cohabitation status at postnatal assessment 1 (2‐6 weeks postpartum) and postnatal assessment 2 (6 months postpartum): group A, mothers who retained custody at both postnatal assessment 1 and postnatal assessment 2; group B, mothers who never lived with their child at either postnatal assessment 1 or postnatal assessment 2; and group C, mothers who lived with their child at only 1 time point (postnatal assessment 1 or postnatal assessment 2). To assess the extent to which psychosocial variables prenatally, postnatally at 2‐6 weeks, and postnatally at 6 months predict custody outcomes, we will use regression modeling as the primary analytical strategy.

Power calculations indicate that a small-to-medium effect size (*f*²=0.25) can be detected with 48 participants in a regression model with 3 predictors (*α*=.05; power=0.80), and a small effect size (*f*²=0.20) can be detected with 52 participants in a model with 2 predictors (*α*=.05; power=0.80). Existing literature supports the relevance of the selected predictors. One study reported significant associations of increased involvement of child protection services with maternal mental health (*χ*²=23.29; *P*<.001), antenatal depression (*χ*²=34.43; *P*<.001), cannabis use (*χ*²=17.90; *P*<.001), and other illicit drug use (*χ*²=24.10; *P*<.001) [74]. Another study found that abstinence during the perinatal period significantly increased the likelihood of custody retention (adjusted *R*²=–1.057; OR 0.35, 95% CI 0.16‐0.77; *P*<.01) [75].

If regression modeling is not feasible due to sample size constraints or violations of statistical assumptions, we will analyze dichotomous variables using log odds ratios derived from questionnaire cutoff scores and custody outcomes. In cases where group comparisons are not viable (eg, due to insufficient numbers in one or more custody categories), qualitative case studies will be conducted to explore individual trajectories and potential predictive factors.

#### Exploratory Analyses

In addition to hypothesis-driven comparisons, we will conduct exploratory analyses to examine potential associations between key variables. These include substance use and postnatal attachment, substance use and mental health, substance use and perceived support, prenatal and postnatal mother-child relationship quality, mental health and postnatal bonding, parental stress and postnatal attachment, and recovery capital and mental health.

#### Qualitative Analyses: Longitudinal TA and Expressed Emotion Coding

All interviews and speech samples will be audio-recorded, transcribed verbatim, and pseudonymized. Semistructured interviews will be analyzed using longitudinal reflexive TA [76], aiming to understand how experiences and meanings related to substance use and recovery, maternal mental health, pregnancy and motherhood, the mother-child relationship, custody/cohabitation, and contextual factors unfold over time.

Given our interest in individual change over time, a trajectory approach within longitudinal reflexive TA will be used [76]. After initial inductive, reflexive coding [77,78], within-case, time-ordered matrices (1 matrix per participant; time on the x-axis, themes on the y-axis) will be constructed to preserve the chronological flow. Subsequently, cross-case trajectory matrices (themes on the y-axis, participants on the x-axis) will be created to compare longitudinal patterns across participants, identifying changes, continuities, and divergent pathways. Matrix development will be iteratively discussed with the broader research team to enhance dependability and transparent decision-making.

The TMSS and FMSS will be analyzed using a validated coding system to assess 2 components of expressed emotion: criticism and EOI [70]. These data will be integrated with interview-based themes to enrich the understanding of the parent-child relationship.

Coding will be iterative and interpretative, and will be supported by MAXQDA. At least two researchers will engage in collaborative, reflexive dialogues to deepen interpretation. Any interpretative differences will be addressed through discussions or, if needed, consultation with a third researcher.

### Integration of Qualitative and Quantitative Findings

Following separate analyses, quantitative and qualitative findings will be integrated within a convergent parallel mixed-methods design using joint displays and narrative weaving [71], aligning quantitative trajectories with qualitative themes at both the case and pattern levels to identify convergence, divergence, and complementarity. This integration will allow us to identify how quantitative patterns align with, extend, or contrast participants’ lived experiences.

### Ethical Considerations

This study received ethical approval from the Medical Ethics Committee of the University Hospital Gent on April 16, 2025 (UZGent; ONZ-2024‐0603). Participants are informed about the confidentiality of the data they provide, and written informed consent is acquired from all participants prior to inclusion in the study. Participants receive a €15 (US $17.47) voucher for each completed assessment. All data are treated confidentially and reported anonymously. This study is conducted in accordance with the Declaration of Helsinki.

## Results

The PhD-study was funded in January 2024. As of March 2025, all surveys have been created and tested in REDCap. Participant recruitment began in May 2025 and is expected to be complete in November 2026. Data collection began in June 2025. As of April 2026, 13 participants have been recruited. Data analysis will be performed after all data have been collected. The results are expected to be published by the end of 2027.

## Discussion

### Anticipated Findings

This protocol describes the design of the PIPPA study, a longitudinal, mixed-methods investigation into the transition to parenthood among women with substance use problems. Drawing on a convergent parallel mixed-methods design [71], the study integrates maternal self-reports and caregiver perspectives to generate an in-depth understanding of the psychosocial and contextual factors shaping short- and long-term maternal and child trajectories.

The PIPPA study is expected to generate insights into how the perinatal period may function as a promising or challenging transition to parenthood for pregnant and parenting women with substance use problems. Drawing on prior research, several anticipated findings may emerge. First, we anticipate substantial heterogeneity in women’s characteristics and substance use patterns across the perinatal period. Recent studies have described distinct classes regarding perinatal substance use and illustrated how pregnant and parenting women in these classes differ in social context, socioeconomic circumstances, and mental health [79]. By capturing this heterogeneity longitudinally and from both maternal and caregiver perspectives, the PIPPA study will clarify how different perinatal substance use trajectories unfold over time. A better understanding of this diversity may inform the development of tailored, responsive, and equitable care approaches for pregnant and parenting women with substance use problems [2,79].

Second, greater severity of substance use is expected to be associated with poorer maternal mental health, lower recovery capital, and less optimal mother-child relationship quality. Prior research links depressive and posttraumatic stress disorder symptoms to increased craving, a higher risk of relapse, and lower prenatal bonding [27,28,80]. Studies have shown that higher substance use severity predicts elevated depressive symptoms postpartum [27]. Recovery capital research has further consistently demonstrated that limited personal, social, and community resources predict worse outcomes and increased relapse risk [45], while supportive networks are protective [81]. Developing greater insights into how these factors relate to and interact with one another across pregnancy and the postpartum period can guide more individualized, context-sensitive support for pregnant and parenting women. Moreover, by examining these factors simultaneously and longitudinally, the PIPPA study can offer a better understanding of how these factors reinforce each other and how women understand and experience the interplay among mental health, substance use, and recovery.

Third, maternal mental health, substance use, and recovery capital are likely to fluctuate across the perinatal period, with many women experiencing the most pronounced distress around 6 months postpartum. This aligns with evidence showing the impact of increased parental stress, limited support, intimate partner violence, identity transition demands, and cumulative vulnerabilities documented among pregnant and parenting women with substance use problems [28,45,82,83]. Prior studies indicate that relapse rates are high during the postpartum period [27-29,83,84], and virtual models predict that by 2 years postpartum, all women who were abstinent during pregnancy resume substance use [28], which may disrupt the mother-child relationship [27]. The longitudinal follow-up in the PIPPA study will make it possible to map how and why needs evolve, providing evidence on long-term needs that have been largely missing from previous, shorter-term studies.

Finally, custody retention and cohabitation outcomes are anticipated to relate strongly to maternal mental health, relational stability, the availability of a supportive network, and substance use patterns. Evidence shows that pregnant and parenting women with substance use problems are at a higher risk of child removal when facing intimate partner violence, unstable housing, untreated mental health difficulties, or polysubstance use [2,29,85,86]. These findings suggest that custody outcomes are shaped less by the mother-child bond itself and more by maternal well-being and contextual stability. By linking maternal well-being, relational stability, recovery capital, and substance use patterns to custody trajectories, this study will help identify which factors primarily shape cohabitation outcomes and which areas of support may meaningfully improve the likelihood of family preservation. Moreover, by tracing diverse trajectories and documenting not only vulnerabilities but also sources of strengths, such as recovery capital and contextual support, the PIPPA study will contribute to a more nuanced and strengths-based understanding of perinatal substance use.

### Dissemination Plan

We plan to disseminate the study findings through peer-reviewed publications, presentations at national and international conferences, and contributions to study days and symposia, thereby strengthening the scientific evidence on perinatal substance use. Additionally, through collaborations with professional networks and interdisciplinary meetings (such as the Perinet expert group on addiction; Vlaams expertisecentrum alcohol en andere drugs [Flemish Center of Expertise on alcohol and other drugs]; and residential mother-child rehabilitation units, including Tipi and Antwerp Drug Intervention Center), the study aims to translate findings into practice-oriented insights for professionals. Furthermore, we will develop accessible summaries tailored for participants and the wider public. These will be shared through involved organizations, care providers, and social media platforms.

### Limitations

Several methodological limitations must be acknowledged. First, the relatively modest sample size may limit statistical power and the ability to detect subtle effects or conduct subgroup analyses. This challenge is amplified by the substantial variability in maternal and child trajectories, which may complicate between-group comparisons. Second, reliance on self-reported measures of substance use and psychosocial variables introduces potential biases, including social desirability and recall biases. Given the stigma surrounding perinatal substance use and concerns about child-welfare involvement, underreporting remains a realistic possibility.

A further limitation concerns the risk of attrition in a population characterized by instability and shifting life circumstances. Although multiple retention strategies are implemented, dropout may still occur and must be considered when interpreting longitudinal outcomes.

### Strengths

Despite these challenges, several features of the PIPPA study strengthen its methodological rigor and interpretative depth. The mixed-methods approach enables the integration of quantitative data with qualitative insights, allowing for meaningful interpretation even when quantitative data are limited or incomplete. Although the expected sample size is modest, it is relatively large for this population and offers a valuable opportunity to explore experiences that are rarely captured in research. The inclusion of women across a broad spectrum of substance use, from ongoing substance use to use in the 6 months before conception, allows for a nuanced understanding of perinatal substance use and highlights opportunities for tailored or less intensive forms of support. Additionally, methodological triangulation, combining quantitative assessments with in-depth interviews of pregnant and parenting women and caregivers, provides comprehensive, multiperspective insights into maternal and child trajectories.

Moreover, this study adopts a strengths-based approach, framing the perinatal period as a window of opportunity [27,37] and focusing on how pregnant and parenting women make meaning of change, pregnancy, and parenthood. This approach moves beyond deficit-oriented perspectives and highlights factors that promote positive transitions to motherhood. The longitudinal design, following pregnant and parenting women from pregnancy through 6 months postpartum, addresses a significant gap in existing research [28].

Lastly, the study’s socioecological engagement and retention strategy [48,49], addressing individual, interpersonal, organizational, community, and policy-level barriers to participation, represents a major methodological strength. This multilayered approach increases feasibility and supports sustained involvement in a population for whom research participation can be particularly challenging [49,50]

### Conclusions

The PIPPA study is designed to advance the understanding of how maternal, psychosocial, and contextual factors shape the transition to parenthood for pregnant and parenting women with substance use problems. By integrating longitudinal quantitative data with maternal narratives and caregivers’ perspectives, this study aims to capture the heterogeneity of women’s experiences and trajectories, and the complex interplay among substance use, mental health, recovery capital, and early parenting. Anticipated findings, such as fluctuations in maternal mental health, variations in substance use and recovery trajectories, and the influence of social support, mirror patterns reported in previous research. The insights generated are expected to contribute to a more nuanced, strengths-based, and evidence-informed understanding of the perinatal period as a promising or challenging transition to parenthood for pregnant and parenting women with substance use problems, which may inform and strengthen integrated and responsive early interventions.

## Supplementary material

10.2196/88030 Multimedia Appendix 1 Interview guide of pregnant and parenting women.

10.2196/88030 Multimedia Appendix 2 Interview guide of caregivers.

## Abbreviations

AUDIT-C: Alcohol Use Disorder Identification Test-Consumption
BARC-10: Brief Assessment of Recovery Capital-10
DUDIT-C: Drug Use Disorder Identification Test-Consumption
EOI: emotional overinvolvement
EPDS: Edinburgh Postnatal Depression Scale
FMSS: Five-Minute Speech Sample task
MAAS: Maternal Antenatal Attachment Scale
MPAS: Maternal Postnatal Attachment Scale
PIPPA: Perinatal Insights into Parenting, Pathways, and Addiction
PSS: Parental Stress Scale
REDCap: Research Electronic Data Capture
SUD: substance use disorder
SWLS: Satisfaction With Life Scale
TA: thematic analysis
TMSS: Three-Minute Speech Sample task
