# Supplementary material for: Perinatal Insights Into Parenting, Pathways, and Addiction (PIPPA): Protocol for a Longitudinal, Strengths-Based Study in Flanders, Belgium
Source: JMIR Res Protoc. 2026 May 21;15:e88030. doi: 10.2196/88030 (PMC13193704; doi:10.2196/88030)
Supplement: Multimedia Appendix 1 [file resprot-v15-e88030-s001.pdf]

Semi-structured interview T1 – Mothers

Well-being of X and the Unborn Child

1. Can you tell me how your pregnancy has been so far?
   (Explore medical health, mental health, changes in mood/mental health, development of the unborn child, etc.)
2. Has your pregnancy changed how you view yourself as a person or your life goals? If so, how?
   (If applicable: Has this pregnancy changed how you view your partner? If so, how?)

(Future) Parenthood

3. What thoughts or feelings do you have about future parenthood/motherhood?
4. What kind of parent do you hope or think you will be?
   (Explore desires/hopes as a future parent; doubts/fears/concerns)
5. How are you preparing for future parenthood?

Substance Use and Pregnancy/Future Parenthood

6. How do you view your substance use in combination with your pregnancy?
   (Explore changes in use since discovering the pregnancy, motivation for change, professional and informal support, concerns/fears about current use, etc.)
   (If applicable: How do you view your partner's substance use in combination with your pregnancy?)
7. How do you view your substance use after giving birth?
   (Explore substance use and parenthood, recovery and parenthood, etc.)

Relationships and Social Environment

8. How does your environment view your pregnancy and (recovery from) substance use?
   (Explore partner, parents, family, friends; support for reducing/continuing substance use; pressure/understanding from others, etc.)
9. Are there specific people, situations, or feelings that have negatively impacted your recovery or well-being, or that currently trigger your use? How do you try to cope with these?
10. Is there anything else you would like to share?

Semi-structured interview T2 – Mothers

Well-being of X and Child

1. Can you tell me how you have been doing so far?
   (Explore delivery, medical health, mental health, changes in mood/mental health, etc.)
2. Can you tell me more about your child's birth?
   (Explore weight, length, head circumference; APGAR score; concerns; neonatal care admission, etc.)
3. Can you tell me more about your child's current health and development? How would you describe your child?
   (Explore weight, length; medical problems/hospitalizations; concerns, extra support, etc.)

Parenthood

4. How are you experiencing motherhood so far? How would you describe yourself as a mother? (Explore resilience, stress; professional and informal support, etc.)

   If child removal occurred: Reasons, child's location, visits, involved agencies, (conditions for) reunification

5. Has the birth of your child changed how you view yourself as a person or your life goals? If so, how?

   If applicable: Has the birth of your child changed how you view your partner? If so, how?

Substance Use and Parenthood

6. How do you view your substance use in combination with parenthood?
   (Explore changes in use since the child's birth, increase/decrease in cravings since birth, motivation for change, professional and informal support, concerns/fears about current use, etc.)

   If applicable: How do you view your partner's substance use in combination with parenthood?)

Relationships and Social Environment

7. How does your environment view your parenthood and (recovery from) substance use?
   (Explore partner, parents, family, friends; support for reducing/continuing substance use; pressure/understanding from others, etc.)
8. Are there specific people, situations, or feelings that have negatively impacted your recovery or well-being, or that currently trigger your use? How do you try to cope with these?
9. Is there anything else you would like to share?

Semi-structured interview T3 – Mothers

Well-being of X and Child

1. Can you tell me how the past six months have been? How have you experienced the first six months of motherhood?
   (Explore medical health, mental health, changes in mood/mental health, etc.)
2. Can you tell me more about your child? How would you describe your child?
3. Can you tell me more about your child's health and development?
   (Explore weight, length; medical problems/hospitalizations; concerns, extra support, etc.)

Parenthood

4. How are you experiencing motherhood so far? How would you describe yourself as a mother? (Explore resilience, stress; professional and informal support, etc.)

   If child removal occurred: Reasons, child's location, visits, involved agencies, (conditions for) reunification

Substance Use and Parenthood

5. How do you currently view your substance use in combination with parenthood?
   (Explore changes in use over the past six months, increase/decrease in cravings since birth, relapse, motivation for change, professional and informal support, concerns/fears about current use, etc.)

   If applicable: How do you view your partner's substance use in combination with parenthood?)

Relationships and Social Environment

6. How does your environment view your parenthood and (recovery from) substance use?
   (Explore partner, parents, family, friends; support for reducing/continuing substance use; pressure/understanding from others, etc.)
7. Are there specific people, situations, or feelings that have negatively impacted your recovery or well-being, or that currently trigger your use? How do you try to cope with these?
8. Is there anything else you would like to share?
