# Supplementary material for: Perinatal Insights Into Parenting, Pathways, and Addiction (PIPPA): Protocol for a Longitudinal, Strengths-Based Study in Flanders, Belgium
Source: JMIR Res Protoc. 2026 May 21;15:e88030. doi: 10.2196/88030 (PMC13193704; doi:10.2196/88030)
Supplement: Multimedia Appendix 2 [file resprot-v15-e88030-s002.pdf]

Semi-structured interview T1- Professional

Relationship with the Mother (referred to as X)

1. Can you describe your relationship with X? How and when did you come into contact with each other?

Well-being of X and the Unborn Child

2. Can you tell me how X is currently doing? (Explore medical health, mental health, substance use)
3. How is the pregnancy progressing? (Explore X's health & follow-up; health and development of the unborn child)

(Future) Parenthood

4. When did you find out that X was pregnant? What were your thoughts/feelings about it?
5. How does X view future parenthood (both for herself and her partner)? What thoughts or feelings does she express about this? (Explore expectations/desires/hopes, fears/doubts/concerns, upbringing experiences)
6. How do you feel about X's (and her partner's) future parenthood?
   (Explore doubts/uncertainties/concerns; strengths and competencies of X (and partner))
   6.1. If potential child removal after birth: Reasons (signs/situations); How are these concerns discussed with the mother? What is her response? Involved agencies/mandated services; What is needed to prevent removal?
7. What steps has she already taken, or has she been helped to take, to prepare for parenthood? How is X's partner involved in care and preparation for parenthood?

Substance Use During Pregnancy and Future Considerations

8. How do you view X's (recovery from) substance use during her pregnancy? How do you view X's (recovery from) substance use and her future parenthood?
   (Explore motivation, partner influence, triggers & cravings, support & barriers, aftercare strategies/plans post-delivery)

Relationships and Social Environment

9. What supportive individuals or networks are currently present in her life?
10. What professional assistance is currently involved? (Explore gaps/barriers, additional necessary support, etc.)
11. Are there people or situations in her environment negatively affecting her recovery or well-being? How do you help her deal with these relationships or situations? (Partner, family, friends, living environment)

Semi-structured interview T2 - Professional

Well-being of X and the Child

1. Can you tell me how X is currently doing? (Explore medical health, mental health, substance use, etc.)
2. Can you tell me more about X's delivery? How was the child at birth? (Weight, length, head circumference, APGAR score, concerns (NAS/FAS/withdrawal symptoms, neonatal care unit stay, etc.))
3. Can you tell me about the current health and development of X's child? How would you describe the child? (Explore weight, length, medical issues/hospitalizations, concerns, extra support)

Parenthood

4. How is X experiencing motherhood so far? (Explore resilience, stress, professional and informal support)

   If child removal occurred: Reasons, child's location, X's visits, impact on X's mental well-being, involved agencies, (conditions for) reunification

5. How would you describe X as a mother? How do you feel about X's (and her partner's) parenthood?
   (Explore doubts/uncertainties/concerns; strengths and competencies of X (and partner))

   If child removal is at risk: Reasons (signs/situations); How are these concerns discussed with the mother? What is her response? Involved agencies/mandated services; What is needed to prevent removal? Are any conditions set (safety plan, childcare, weekly testing, follow-up with drug counseling, etc.)?

Substance Use and Parenthood

6. How do you view X's (recovery from) substance use and her parenthood? How do you view X's partner's (recovery from) substance use and parenthood?
   (Explore concerns, X's motivation, changes in substance use since childbirth, partner's use, triggers & cravings, support & barriers, aftercare strategies/plans, relapse, combining childcare and substance use)

Relationships and Social Environment

7. What supportive individuals or networks are currently present in her life?
8. What professional assistance is currently involved? (Explore barriers in finding/accessing help, additional necessary support, collaboration between involved agencies)
9. Are there people or situations in her environment negatively affecting her recovery or well-being? How do you help her deal with these relationships or situations? (Partner, family, friends, living environment)

Semi-structured interview T2 - Professional

Well-being of X and the Child

1. Can you tell me how X is currently doing? (Explore medical health, mental health, substance use, etc.)
2. Can you tell me about the current health and development of X’s child? How would you describe the child? (Explore weight, length, medical issues/hospitalizations, concerns, extra support)

Parenthood

3. How is X experiencing motherhood so far? (Explore resilience, stress, professional and informal support)

   If child removal occurred: Reasons, child’s location, X’s visits, impact on X’s mental well-being, involved agencies, (conditions for) reunification

4. How would you describe X as a mother? How do you feel about X’s (and her partner's) parenthood?
   (Explore doubts/uncertainties/concerns; strengths and competencies of X (and partner))

   If child removal is at risk: Reasons (signs/situations); How are these concerns discussed with the mother? What is her response? Involved agencies/mandated services; What is needed to prevent removal? Are any conditions set (safety plan, childcare, weekly testing, follow-up with drug counseling, etc.)?
5. How has parenthood potentially changed X (and her partner)? (Positive, negative, adjustments to the role)

Substance Use and Parenthood

6. How do you view X's (recovery from) substance use and her parenthood? How do you view X's partner's (recovery from) substance use and parenthood?
   (Explore concerns, X’s motivation, changes in substance use since childbirth, partner's use, triggers & cravings, support & barriers, aftercare strategies/plans, relapse, combining childcare and substance use)

Relationships and Social Environment

7. What supportive individuals or networks are currently present in her life?
8. What professional assistance is currently involved? (Explore barriers in finding/accessing help, additional necessary support, collaboration between involved agencies)
9. Are there people or situations in her environment negatively affecting her recovery or well-being? How do you help her deal with these relationships or situations? (Partner, family, friends, living environment)
